# Site-Specific Incorporation of Unnatural Amino Acids into Escherichia coli Recombinant Protein: Methodology Development and Recent Achievement

**DOI:** 10.3390/biom9070255

**Published:** 2019-06-28

**Authors:** Sviatlana Smolskaya, Yaroslav A. Andreev

**Affiliations:** 1 Sechenov First Moscow State Medical University, Institute of Molecular Medicine, Trubetskaya str. 8, bld. 2, Moscow 119991, Russia; 2 Shemyakin-Ovchinnikov Institute of Bioorganic Chemistry, Russian Academy of Sciences, ul. Miklukho-Maklaya 16/10, 117997 Moscow, Russia

**Keywords:** noncanonical amino acids, expanded genetic code, orthogonal aminoacyl-tRNA synthetase (aa-RS)/tRNA pair, nonsense suppression

## Abstract

More than two decades ago a general method to genetically encode noncanonical or unnatural amino acids (NAAs) with diverse physical, chemical, or biological properties in bacteria, yeast, animals and mammalian cells was developed. More than 200 NAAs have been incorporated into recombinant proteins by means of non-endogenous aminoacyl-tRNA synthetase (aa-RS)/tRNA pair, an orthogonal pair, that directs site-specific incorporation of NAA encoded by a unique codon. The most established method to genetically encode NAAs in *Escherichia coli* is based on the usage of the desired mutant of *Methanocaldococcus janaschii* tyrosyl-tRNA synthetase (*Mj*TyrRS) and cognate suppressor tRNA. The amber codon, the least-used stop codon in *E. coli*, assigns NAA. Until very recently the genetic code expansion technology suffered from a low yield of targeted proteins due to both incompatibilities of orthogonal pair with host cell translational machinery and the competition of suppressor tRNA with release factor (RF) for binding to nonsense codons. Here we describe the latest progress made to enhance nonsense suppression in *E. coli* with the emphasis on the improved expression vectors encoding for an orthogonal aa-RA/tRNA pair, enhancement of aa-RS and suppressor tRNA efficiency, the evolution of orthogonal EF-Tu and attempts to reduce the effect of RF1.

## 1. Introduction

Only twenty amino acids composing huge repertoire of proteins with the remarkably diverse structure and function are encoded by the universal genetic code of all known organisms [1] with the rare exceptions of selenocysteine being incorporated in response to the stop codon UGA [2,3] and pyrrolysine specified by the UAG codon [3,4]. Obviously, 20 amino acid building blocks are sufficient for life, but not optimal, since proteins’ natural functions are provided by chemical groups obtained through posttranslational modifications (i.e., phosphorylation, methylation, acetylation, and hydroxylation); cofactors (such as pyridoxal, thiamine, flavins, and metal ions); and, as mentioned before, by evolved novel translational machinery to incorporate either selenocysteine or pyrrolysine [5,6].

Given the important roles of proteins in biology, it is desirable to be able to rationally modify their structure and function to aid understanding of protein structure-function relationships, investigate protein-based biological processes, and generate proteins and organisms with new properties [7]. For the protein chemists, it would be ideal to use amino acids with novel chemical or spectroscopic properties, i.e., unnatural or noncanonical amino acids (NAAs) [8].

Many diverse techniques were developed for the incorporation of NAAs into proteins to introduce new functional groups apart from those found in the canonical amino acids including chemical approach based on direct modification or synthesis of the desired proteins [9,10,11], and biosynthetic methods to totally replace canonical amino acid to their close structural analogues in auxotrophic bacteria [12,13,14,15] or co-translationally incorporate NAAs into target proteins by utilizing chemically modified aminoacylated tRNA molecules for in vitro translation [16,17,18,19] or for *Xenopus* oocytes [20,21,22]. Although these approaches for the generation of NAA-incorporated proteins are extremely useful for altering protein structure and properties and can be applied for a large variety of NAAs, their application is limited by the selectivity and overall efficiency of the methodology. 

The general methodology of the genetic code expansion is based on the reassignment of either sense or nonsense codon to encode NAA. This methodology for NAAs incorporation exploits the translational machinery of the host cell in the same manner as the canonical amino acids. More than 200 NAAs with diverse physical, chemical, or biological properties have been incorporated into proteins in vivo and in vitro in *E. coli* and other bacteria, yeast, animals and mammalian cells with high fidelity and efficiency by genetic code expansion [23]. Here we are focused on the description of the methodology and the recent progress in the field of *E. coli* genetic code expansion with the emphasis on the nonsense suppression, since to date, it is the most robust, established and demanded techniques for the recombinant protein production.

## 2. Expanding the Genetic Code 

An ideal methodology to incorporate NAAs into recombinant proteins was considered to exploit the translational machinery of the host cell in the same manner as the canonical amino acids, enables specific changes to be precisely made in proteins directly in vivo, thus providing novel tools for understanding biology in molecular terms in the native settings. In light of the large body of knowledge about its translational machinery and the simplicity of its genetic manipulation, *E. coli* was chosen as the host organism to develop the general method to co-translationally incorporate NAAs in vivo. 

**Figure 1:**
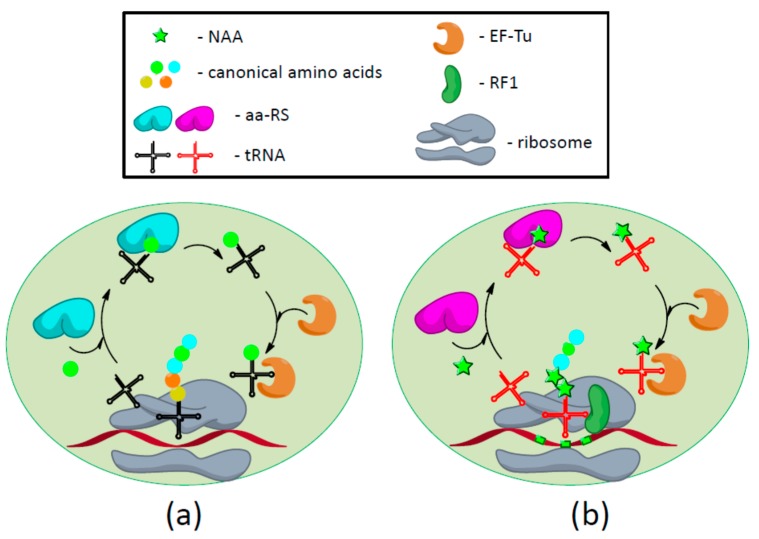
Schematic representation of expanded genetic code methodology. (**a**) Translation in *E. coli* cell: Endogenous aminoacyl-tRNA synthetases (aa-RS) (designed by cyan) charge tRNA (black colour) with cognate amino acids and aminoacyl-tRNA is transferred by elongation factor Tu (EF-Tu) to the ribosome. Proper interaction of mRNA codon and tRNA is required for the release of EF-Tu and peptide bond formation; (**b**) the methodology of genetic code expansion: NAA incorporation in response to amber stop codon is provided by supplying the organism with orthogonal aa-RS (violet colour) and suppressor tRNA (red colour). Suppressor tRNA is in competition with class I release factor (RF1).

Process of translation is the ribosome-mediated production of a polypeptide in which the amino acid sequence is specified by the nucleotide sequence of the mRNA. Nucleotides form 64 three-base codons or triplets that encode a total of 20 amino acids. Three of the 64 codons, UAG (amber), UGA (opal), and UAA (ochre), are termination codons, alternatively called stop or nonsense codons. mRNA serves as a template for the synthesis of a polypeptide chain through a tRNA adapter molecule charged with an amino acid corresponding to its anticodon. The charging of each amino acid is carried out by specific aminoacyl-tRNA synthetase (aa-RS) forming aminoacyl-tRNA (aa-tRNA). Consequently, each aa-RS is specific for its own unique amino acid substrate, and a unique set of iso-tRNAs exists for each amino acid [24,25]. Although tRNA anticodons are conserved across all organisms, aa-RSs are often species-specific; that is, aa-RSs from one species do not aminoacylate tRNAs from other species. In the first step of translation, initiation, the small ribosomal subunit (30S) is associated to an mRNA through the correct base pairing of Shine–Dalgarno sequence (SD) with 16S ribosomal RNA (rRNA). Subsequently, the initiator aminoacyl-tRNA, formylmethionine-tRNA, binds with the help of initiation factors 1 (IF1) and 2 (IF2) to the AUG start codon of mRNA that, in turn, leads to the association with the 50S ribosomal subunit with the complex. Formylmethionine-tRNA is bound to the P (peptidyl) site of the now 70S ribosome, with the second codon displayed in the available A (aminoacyl) site. The series of events is partly driven by the hydrolysis of IF2 bound GTP. During the second phase, elongation, aminoacyl-tRNA in ternary complex with elongation factor Tu (EF-Tu) and GTP binds to the A site of the 70S ribosome. Correct codon-anticodon recognition causes hydrolysis of GTP bound to EF-Tu, inducing its dissociation and the proper accommodation of the aa-tRNA into the A site. A peptide bond between the newly arrived amino acid and the formylmethionine is formed in the catalytic site of the 50S subunit formation, thereby generating a dipeptide, which is then transferred from the P-site tRNA to the amino acid on the A-site. EF-Tu is recycled by the rapid exchange of GDP to GTP, facilitated by elongation factor Ts (EF-Ts). The translocation step is facilitated by elongation factor G (EF-G) and the hydrolysis of another GTP molecule. The elongation cycle proceeds until a stop codon in the mRNA is encountered, which triggers recruitment of a class I release factor (RF1 or RF2 depending on the stop codon) to the A site of the ribosome. The RF-ribosome interaction leads to hydrolysis of the ester bond between the C-terminal amino acid of the polypeptide and the P-site tRNA, releasing the polypeptide from the ribosome. The GTP associated class II release factor 3 (RF3) then destabilizes the class I release factor and they both dissociate, leaving the 70S ribosome bound to mRNA and a now deacylated tRNA in P site. In the final step, the ribosome is split by the ribosome recycling factor (RRF) and EF-G·GTP and held apart ready for another round of protein synthesis [26] (Figure 1a). 

As is the case with canonical amino acids, NAA can be incorporated by supplying organisms with a non-endogenous aa-RS/tRNA pair, referred to as an orthogonal pair that directs site-specific incorporation of NAA in response to a unique codon [6]. This aa-RS/tRNA/codon set must not crosstalk with endogenous aa-RS/tRNA/codon sets (i.e., be orthogonal) and must be functionally compatible with other components of the translation apparatus [27]. Specifically, the orthogonal tRNA should not be recognized by any endogenous synthetase and should decode the orthogonal codon, which is not assigned to any canonical amino acid. The orthogonal synthetase should not charge any endogenous tRNA but the orthogonal tRNA. In addition, the orthogonal synthetase should only charge the orthogonal tRNA with the NAA. The NAA must be metabolically stable and have good cellular bioavailability; it must be tolerated by EF-Tu and the ribosome, but it cannot be a substrate for any endogenous synthetase [5]. Once expressed in cells, the orthogonal synthetase charges the orthogonal tRNA with the desired NAA, and the acylated tRNA incorporates the attached NAA into proteins in response to the unique codon by utilizing the endogenous translational machinery [28] (Figure 1b). 

**Figure 2:**
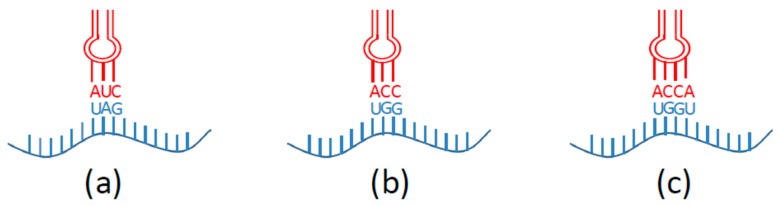
Scheme of nonsense (**a**), sense (**b**) and frame-shift (**c**) suppression demonstrates the interaction of mRNA with orthogonal tRNA.

A unique codon is required to specify the NAA. Initially, site-specific incorporation of NAA into a protein was based on nonsense suppression (Figure 2a) [16,29]. The term “nonsense suppression” refers to the use of nonsense codons and suppressor tRNAs, which recognize stop codons. The amber UAG stop codon has been most frequently used for the incorporation of NAAs, but the use of the ochre (UAA) stop [30,31,32] and the opal (UGA) stop codons [33,34,35] have been applied, especially for the incorporation of diverse NAAs into one recombinant protein [36,37,38,39]. The major advantage of the nonsense suppression technique lies in its simplicity. Nevertheless, even amber stop codon terminates about 320 genes in the model laboratory strain of *E. coli* K-12 [40,41], meaning that the NAA would be incorporated not only in the protein of interest but also in response to TAG stop codons of the genes present in the genome.

The existence of only three stop codons in the genetic code limits the theoretical number of NAAs that could be incorporated into recombinant proteins. Sisido and coworkers suggested overcoming this limitation with frameshift suppression based on the application of four- or five-base extended codon and a cognate suppressor [42,43,44]. In certain species, some naturally occurring codons are rarely used and the amount of their corresponding tRNA is low. Rarely used sense codons, such as arginine (AGG) [45,46] or proline (CCC) codons of *E. coli*, can be used for direct NAA incorporation in proteins [47] or for designing of extended codons and frameshift suppression (Figure 2b,c) [44]. In this approach, an mRNA containing an extended codon consisting of four or five bases is read by a modified aa-tRNA containing the corresponding extended anticodon, and a full-length protein containing NAA at the specific site is obtained. If the extended codon is read as a three-base codon by an endogenous tRNA, the reading frame will be shifted by one base that in turn leads to a premature encounter with a stop codon and early termination of protein synthesis [26].

In each case, suppression is in competition with other processes, such as RF binding to nonsense codons or recognition of frameshift codons by tRNAs with three-base anticodons, both of which lead to decreased co-translational incorporation efficiency [5].

## 3. Orthogonal Pairs for NAAs in *E. coli*

The generation of orthogonal aa-RS/tRNA pairs derived from the translation apparatus of the host organism was challenging, considering that there is already a pool of aa-RS/tRNA pairs assigned for their cognate codons presented within cells. A successful strategy was to import an aa-RS/tRNA pair from a different species. Archaeal synthetases/tRNAs have been considered as a good source of orthogonal aa-RS/tRNA pairs to apply in *E. coli*. One of the reasons is that although synthetases from archaea are phylogenetically closer to their eukaryotic counterparts [24,25], they can be expressed in *E. coli* in their active forms. Moreover, cross-species aminoacylation of archeal tRNA by *E. coli* synthetases has been shown to be low [48]. The first orthogonal aa-RS/tRNA pair in *E. coli* generated from archaeal bacteria was derived from the tyrosyl-tRNA synthetase (TyrRS)/tRNA^Tyr^ from *Methanocaldococcus jannaschii* [49]. It was known that amber stop codon was the least used codon and the presence of natural amber suppressors in some *E. coli* strains did not significantly affect cell-growth rates [50,51], hence, amber was initially applied to specify the NAA. Additionally, chemically aminoacylated amber suppressor has been used for the in vitro introduction of NAAs into proteins in *E. coli*-based cell-free system [52]. *M. jannaschii* tRNA^Tyr^ recognition elements include bases A73 and C1-G72 in the acceptor stem, while the anticodon triplet participates only weakly in identity determination [53]. Since *E. coli* tRNA^Tyr^ identity determination pattern differs from that of *M. jannaschii* and additionally includes a long variable arm, and the anticodon as identity elements, *Mj*tRNA^Tyr^ was considered to be a good candidate for the orthogonal suppressor. Additionally, *Mj*TyrRS has a minimalist anticodon-loop-binding domain [54], which made it possible to alter the anticodon loop of *Mj*tRNA^Tyr^ to CUA with a relatively low reduction in its affinity for the synthetase [6]. Finally, *Mj*TyrRS does not have an editing mechanism that could deacylate the NAA [55]. The *M. jannaschii* tRNA^Tyr^_CUA_ (*Mj*tRNA^Tyr^_CUA_) with mutated anticodon for recognizing the amber codon UAG and its cognate *Mj*TyrRS functioned efficiently in translation in *E. coli*, but there was a small degree of aminoacylation of this *Mj*tRNA^Tyr^_CUA_ by endogenous *E. coli* synthetases [49].

To improve the orthogonality of *Mj*tRNA^Tyr^_CUA_ a general strategy was developed, consisting of a combination of negative and positive selections with a library of tRNA mutants in the absence and presence of the cognate synthetase, respectively [56]. Firstly, 11 nucleotides that do not interact directly with *Mj*TyrRS were randomly mutated to generate a suppressor-tRNA library. Then suppressors were expressed in *E. coli* along with a toxic ribonuclease barnase gene, with amber nonsense codons introduced into the permissive sites. *Mj*tRNA^Tyr^_CUA_ variants which were substrates for endogenous *E. coli* aa-RSs (i.e., were not orthogonal) were removed from the library by suppression of amber nonsense mutations in the barnase gene, resulting in cell death. All tRNAs from surviving clones were then subjected to a positive selection in the presence of the cognate heterologous synthetase and a β-lactamase gene with an amber codon at a permissive site. An orthogonal variant of *Mj*tRNA^Tyr^_CUA_ was selected on the basis of its ability to be aminoacylated by its cognate *Mj*TyrRS and to suppress the amber codon to confer ampicillin resistance [6,57]. Therefore, selected *Mj*tRNA^Tyr^_CUA_ suppressor was not a substrate for the endogenous *E. coli* synthetase, but only for its cognate *Mj*TyrRS; and it functioned efficiently in translation.

A similar two-step selection scheme was then used to alter the specificity of the orthogonal synthetase to charge its cognate *Mj*tRNA^Tyr^_CUA_ suppressor with unique NAA of interest and none of the canonical amino acids. First, a library of aa-RS mutants containing randomized residues in the amino acid-binding site was constructed on the basis of available crystal structures [58,59]. This library was transformed into *E. coli* cells that express tRNA_CUA_ and a gene encoding chloramphenicol acetyltransferase (CAT) with a stop codon mutation at a permissive site. In this positive selection step, these cells were grown in the presence of chloramphenicol and the NAA of interest so that only the aa-RS mutants capable of aminoacylating tRNA_CUA_ with the NAA and/or endogenous amino acids survived. Surviving mutants were then transformed into *E. coli* cells that express tRNA_CUA_ and the toxic barnase gene with stop codon mutations at permissive. In this negative selection step, cells were grown in the absence of the NAA so that all clones whose mutant aa-RS aminoacylates endogenous amino acids died. Only mutant aa-RSs that aminoacylate tRNA_CUA_ with the NAA left. The two-step selection is usually repeated for two to three additional rounds to yield NAA-specific mutant aa-RS. This two-step selection scheme was applied for the identification of mutant orthogonal TyrRS/tRNA^tyr^ pairs with altered substrate specificity. *M. jannaschii* synthetase/tRNA pairs characterized by excellent fidelity have been applied for incorporation of chemically distinct NAAs into recombinant proteins of *E. coli* [5], *Salmonella* [60], *Streptomyces venezuelae* ATCC 15439 [61] and *Mycobacterium tuberculosis* [62].

Nevertheless, the ability to encode novel NAAs is determined and limited by the structure of the synthetase active site. In order to expand the repertoire and diversity of NAAs, several additional orthogonal pairs have been adapted for application in *E. coli*. These pairs include some derived from archaea, such as a lysine-RS/tRNA^Lys^ pair from *Pyrococcus horikoshii* [63], a glutamine-RS/tRNA^Glu^ pair from *Methanosarcina mazei* [64], pyrrolysine-RS/tRNA^Pyl^ pair from *Methanosarcina* species [65,66,67]; as well as a heterologous pair consisting of a leucyl-tRNA synthetase from *Methanobacterium thermoautotrophicum* and a mutant tRNA^Leu^ derived from *Halobacterium sp*. [34], and pair consisting of *P. horikoshii* proline-RS and three engineered suppressors tRNA^Pro^ from *Archaeoglobus fulgidus* [68].

### 3.1. Optimized Orthogonal Translation System for NAA Incorporation into Recombinant Proteins in E. coli

Nowadays, genetic code expansion in *E. coli* using the amber suppression strategy and evolved variants of orthogonal *M. jannaschii* TyrRS/*Mj*tRNA^Tyr^_CUA_ is considered to be the most established and robust methodology to site-specifically incorporate NAAs. However, the number of NAA-incorporated proteins obtained with orthogonal *M. jannaschii* aa-RS/*Mj*tRNA pairs in vivo is considerably lower in comparison to the wild type. One of the reasons for the low yields of NAA-incorporated protein in the nonsense suppression studies is that both orthogonal synthetase and its suppressor tRNA are transferred into the host organisms and presented in the ratio that is not optimal for the efficient functioning in the translational process. The most fundamental improvements were based on the development of the vector system for the optimal expression of recombinant proteins. The significant increase in NAA-containing protein yield was achieved by the replacement of two plasmids, each one encoding either synthetase or suppressor tRNA, to the single-plasmid system pSup-*Mj*TyrRS-6TRN [69]. The plasmid backbone belongs to the most popular expression ColE1-like plasmid vectors [70] with a mid-copy-number to low-copy-number p15A origin that makes it compatible with most of the vectors designed for protein expression in *E. coli* background. The designed pSup-*Mj*TyrRS-6TRN vector allowed increasing the NAA-containing protein yield due to the number of regulatory elements modification: Firstly, the amber suppressor tRNA is expressed under regulation of naturally occurring *E. coli* prolyl-tRNA promoter and terminator (*proK*), the plasmid encodes six copies of *Mj*tRNA^Tyr^_CUA_ in two polycistronic operons; secondly, *E. coli* glutaminyl-tRNA synthetase promoter (*glnS*) regulating expression of the orthogonal variants of *M. jannaschii* TyrRS was mutated to *glnS’* promoter [71]; finally, the single mutation D286R was introduced in the sequence of *Mj*TyrRS and its derivatives that led to the enhanced recognition of anticodon of *Mj*tRNA^Tyr^_CUA_ suppressor [58]. The yield of purified NAA-incorporated proteins obtained due to the application of pSup-*Mj*TyrRS-6TRN plasmid system has been reported to reach 40 mg/L.

The plasmid vector pEVOL was reported to afford enhanced NAA incorporation into recombinant proteins in comparison with pSup-*Mj*TyrRS-6TRN. It is known that suppression efficiency depends greatly on the degree of orthogonal tRNA compatibility with the translational apparatus of the host cell. The reasons for such an incompatibility are the low affinity of *E. coli* EF-Tu to NAA-charged *Mj*tRNA_CUA_ derived from archeal tRNA^Tyr^ [72] or its inability to recognize and deliver orthogonal tRNA to ribosomal A-site [73]. A number of genetic, biochemical, and structural studies have implicated specific residues within the tRNA acceptor stem and T stem as being important for EF-Tu binding [72]. Directed evolution experiments revealed that GC-rich T-stem of *Mj*tRNA allowed identifying the modified suppressor tRNA_CUA_^opt^ that increased NAA incorporation efficiency due to enhanced affinity to EF-Tu of *E. coli* [74]. The higher yield of mutant protein obtained with the use of the plasmid pEVOL was achieved through the application of the T-stem modified suppressor tRNA_CUA_^Opt^ expressed under the regulation of *proK* promoter combining with the use of both constitutive (*glnS’*) and arabinose-inducible (*araBAD*) promoters to drive the transcription of two copies of the *M. jannaschii* aa-RS gene. Hence, yields were further increased to 100 mg/L and were around 250% greater than the previously developed pSup-*Mj*TyrRS-6TRN system, ranging from roughly 35% to 50% of wild-type protein for most mutant aa-RS/tRNA pairs [75].

An important role of orthogonal tRNA modification for overall suppression efficiency was further demonstrated [76] by the evolution of *Mj*tRNA^Cys^ with altered anticodon-flanking regions for phosphoserine incorporation. The library of suppressor tRNAs molecules with random nucleotide combination in G29-U33 and G37-C41 that flank the anticodon was subjected to the selection pressure. The application of selected variants *Mj*tRNA^Cys^ with enhanced suppression efficiency in combination with evolved *Methanococcus maripaludis* SepRS with modified anticodon binding domain enabled site-specific incorporation of phosphoserine into recombinant proteins. Although the progenitor orthogonal pair consisting of *M. maripaludis* SepRS and *Mj*tRNA^Cys^ enabled NAAs incorporation only in the presence of evolved EF-Tu [73], several evolved *Mj*tRNA^Cys^ variants did not require EF-Tu modification. 

Further enhancement of the yield of recombinant proteins with site-specifically incorporated single or multiple NAA was achieved by the application of pUltra plasmid due to plasmid backbone and regulatory elements substitutions [77]. In contrast with pSup-*Mj*TyrRS-6TRN and pEVOL vector systems, this one utilizes pCDF-1b plasmid backbone with the *Clo*DF13 replicon (*cdf*), spectinomycin resistance cassette and *lacI*-regulatory elements. The application of the high-copy-number *cdf* origin of replication provided higher expression level of orthogonal pair. On the other hand, *cdf* is a rare ori [78] that in combination with spectinomycin resistance cassette made pUltra compatible with another commonly used plasmids. The pUltra plasmid encoded one copy of *M. jannaschii* aa-RS under the regulation of hybrid *tacI* promoter inducible by IPTG, the coding sequence of aa-RS was flanked by a strong ribosome binding site sequence (AAGGAG) and rrnB transcription-termination sequence from pEVOL plasmid at 5′- and 3′-ends, respectively. In similarity with pEVOL plasmid, a single copy of optimized tRNA_CUA_^Opt^ under the regulation of *proK* promoter was used in pUltra plasmid. The application of developed plasmid provided faster expression kinetic and higher suppression efficiency (20%–50% and 200%–300% higher for the suppression of 1 and 3 amber stop codon, respectively, compared with the pEVOL). This plasmid was shown to be useful for the expression of weak orthogonal pairs [68] for the enhanced ochre nonsense suppression by optimized *Methanosarcina* PylRS/tRNA^Pyl^ pair and for the efficient incorporation of two distinct NAAs applying different orthogonal aa-RS/tRNA pairs for amber and ochre suppression encoded in the same plasmid [77]. 

However, the replacement of multiple copies of *M. jannaschii* orthogonal synthetase to the single copy inserted into bacterial chromosome demonstrated [79] that evolved aa-RS was an inefficient enzyme and, hence, an improved suppression efficiency achieved by applying pEVOL and pUltra plasmid was based on the enhanced level of orthogonal pairs expression or multiplication of gene copies number in expression vector. Very recently, the efficiency of NAA incorporation by *M. jannaschii* orthogonal *Mj*TyrRS/*Mj*tRNA^Tyr^_CUA_ pair and its derivatives in response to stop codon was enhanced by further evolution of orthogonal synthetase to improve the catalytic activity toward cognate NAAs. Since the anticodon of *M. jannaschii* tyrosine-tRNA was altered from GUA to CUA, the *Mj*TyrRS recognition of *Mj*tRNA^Tyr^_CUA_ became less effective. Guo et al. [80] applied a directed evolution approach. Briefly, the thorough analysis of the crystal structure of *Mj*TyrRS revealed four amino acids residues (Phe261, His283, Met285, and Asp286) in anticodon recognition pocket responsible for interaction with *Mj*tRNA^Tyr^_CUA_; these amino acids were randomly mutated by means of overlapping PCR and the resulting aa-RS library was subjected to two or three rounds of positive-negative selection. The positive selection revealed functional synthetase by their ability to suppress stop codon in CAT Asp112Stop in the presence of NAA, while the negative selection in the absence of NAA eliminated non-specific aa-RS charging suppressor tRNA by canonical amino acids leading to the expression of toxic barnase gene with amber stop codon at permissive sites. The direct evolution approach was successfully used to select improved variants of mutated *M. jannaschii* p-acetyl-L-phenylalanyl-tRNA-synthetase (AcRS) [81], p-benzoyl-L-phenylalanyl-tRNA-synthetase (BpaRS) [82], and sulfotyrosyl-tRNA-synthetase (sTyrRS) [83]. The evolved variants of *M. jannaschii* orthogonal synthetases demonstrated a similar pattern of mutation, however, the mutation combination differed for every aa-RSs. The observed improvement resulted from more efficient aminoacylation of *Mj*tRNA^Tyr^_CUA_ by the evolved *Mj*TyrRS mutants. Another approach to improve the enzyme activity of *M. jannaschii* orthogonal synthetase was suggested by Amiram et al. [79]. First, genes encoding for the orthogonal AcFRS/tRNA_CUA_^Opt^ system [75] and four amber codon-containing TolC membrane channel for colicin ColE1 translocation were integrated into the intergenic region of the bacterial chromosome. Resulting organisms were used for the generation of aa-RSs library by diversification by multiplex automated genome engineering (MAGE) [84] using single-stranded DNA (ssDNA) pool designed for the mutagenesis of selected amino acids. Obtained diversified cell pool was subjected for the negative selection in the presence of colicin Col E1 to eliminate not orthogonal aa-RS; the remaining orthogonal AcFRS library went through the positive selection in the presence of NAA to isolate variants with improved enzyme efficiency. This approach was used to generate mutated synthetases with improved amino acid binding pocket and modified tRNA binding interface. The suppression efficiency of generated variants of AcFRS varied and was estimated to be 5-25-fold higher compared with the progenitor enzyme, and enhanced enzyme efficiency allowed incorporation of 30 NAAs in the elastin-like peptide, ELP. Notably, that described methodology was also applied for the evolution of aa-RS variants with tunable specificity allowing for the incorporation of 14 NAA. Another alternative to the traditional directed evolution approach that enables fast production of highly effective and selective orthogonal aa-RS is based on phage-assisted continuous evolution PACE selection [85]. PACE technology utilizes mutagenesis plasmids encoding for the variety of genes affecting replication fidelity that provides high mutagenesis efficiency and broad mutational spectrum [86]. Improved variants of aa-RS were selected by their ability to suppress amber stop codon at permissive positions of T7 RNA polymerase, and bacteriophage protein III, which is required for phage infections, or a dominant-negative variant of protein III as a basis for the positive and negative selection, respectively. PACE application enables to combine positive and negative selection steps since there is no need for plasmids isolation; thus only 48 h required for the evolution of improved variant of *M. jannaschii* polyspecific orthogonal synthetase. The highly effective chimeric *Methanosarcina* spp. pylRS selected by PACE demonstrated 45-fold higher enzymatic activity compared to progenitor enzymes.

In addition to the optimization of vector systems and orthogonal synthetase/tRNA themselves, the EF-Tu factor can be mutated for the better recognition of suppressor tRNA charged with NAAs. Although different tRNAs aminoacylated by their cognate natural amino acids have a comparable affinity to EF-Tu, the efficiency of EF-Tu binding is lower for mis-acylated tRNAs, as well as for the suppressor tRNAs charged with NAAs [72]. It was possible to enhance the affinity of *E.* *coli* EF-Tu to NAAs with large side chain by means of enlarging the aminoacyl-binding pocket. The application of EF-Tu mutated variants in an *E. coli*-base in vitro translation system allowed increased efficiency of NAA incorporation with a large aromatic side chain or altered backbones [87,88]. The strategy to enlarge binding pocket of EF-Tu based on the substitution of 10 amino acids to smaller alanine followed by the screening of most efficient mutant was applied [89]: The selected variant of mutated EF-Tu (S65A, D216A, and V274A) demonstrated 4-fold improvement in suppression efficiency. The orthogonal tRNA aminoacylated by negatively charged NAAs, such as NAAs with a phosphate group, cannot be bound and effectively transported by *E.* *coli* EF-Tu. An evolved by random mutagenesis variant of EF-Tu with altered binding pocket was applied for *in vivo* incorporation of phosphoserine along with orthogonal heterologous pair consisting of *M. maripaludis* SepRS and *Mj*tRNA^Cys^ [73]. Another mutant of EF-Tu with substitution of E216V, D217G, F219G amino acids in the binding pocket was rationally designed and successfully applied for the efficient incorporation of *O*-phosphotyrosine alongside with selected mutant of *M. jannaschii* TyrRS and tRNA^Tyr^_CUA_ [90].

Another successful strategy for the suppression efficiency enhancement was based on the creation of orthogonal ribosome/mRNA pairs in *E. coli*. This orthogonal ribosome (o-ribosome)/orthogonal mRNA (o-mRNA) pair was selected and introduced into the *E. coli* host organism. Since the SD sequence of mRNA along with the compliment anti-Shine-Dalgarno (ASD) sequence of 16S rRNA of ribosome is mutated, this pair functions in parallel with, but independent of the endogenous ribosome/mRNA pairs, i.e., o-ribosome exclusively translates the orthogonal mRNA, and the orthogonal mRNA is not a substrate for cellular ribosomes [91]. The evolved o-ribosome was applied to develop modified A-site of 30S ribosome enabling more efficient NAAs incorporation in response to AUG (ribo-*X*) [92] and AUG [93] stop codon, frame-shift AAGA and AGGA codons (ribo-*Q*) [66]. The P-site of 50S ribosome was also modified. Following several rounds of random mutagenesis and selection obtained the 23S rRNA that was applied for *in vitro* incorporation of D- [94] and β-amino acids [95,96]. Another orthogonal 23S rRNA was developed for efficient recognition of the 3′-terminal end of orthogonal tRNA [97]. Due to the continuous association-dissociation reaction of ribosomal subunits, it was not possible to combine evolved 16S and 23S subunits to additionally enhance the efficiency of NAAs incorporation. However, very recently the orthogonal ribosome technology stopped to be limited by only 30S or 50S subunit of ribosome due to the construction of the tethered [98] and stapled [99] ribosome with linked 16S and 23S rRNA. 

### 3.2. Enhancing suppression efficiency by RF1 level manipulation

One of the most important factors affecting suppression efficiency is the competition between suppressor tRNA with RF for binding to the stop codons. Prokaryotic RF functionality depends on the particular stop codon encoded in the mRNA sequence, i.e., RF1 is specific for UAA/UAG, and RF2 is specific for UAA/UGA [100]. Different approaches have been described to increase suppression efficiency of amber suppressor tRNAs via manipulation of RF1 activity, such as the use of *E. coli* strains with thermosensitive RF1 variants [101] and RF1-depleted *E. coli* strains [102,103]. Unfortunately, the *prfA* gene encoding the RF1 is essential for survival; hence, *prfA* gene deletion, which encodes RF1, was reported to be lethal in *E. coli* [104]. However, significant progress was made recently towards diminishing the competition with RF1 by either evolution of orthogonal ribosome or by new approaches to RF1 deletions.

One of the methodologies to enhance the incorporation of NAAs in vivo by minimizing the effects of RF1 relies on selective binding of suppressor tRNA by ribosomal A-site. As it is mentioned above, the o-ribosome)/o-mRNA pair was developed for efficient NAA-containing protein expression [91]. Next, this o-ribosome was used for the creation of the library with random mutations in the 530 loop of the decoding centre of 16S rRNA and selection of mutant with an enhanced ability to decode UAG stop codon on o-mRNA with orthogonal suppressor tRNA [92]. This evolved orthogonal ribosome (ribo-*X*) facilitates site-specific NAAs incorporation in response to amber stop codon by decreasing RF1 binding. 

Although the total removal of the *prfA* gene was known to be lethal for *E. coli* cells, the idea of creating RF1-deficient strains with an enhanced ability for site-specific NAA incorporation was very attractive. The early progress was based on the construction of *E. coli* strains with a temperature-sensitive RF1, which was almost completely inactive at 37 °C [104,105]. These mutants were characterized by increased efficiency of the amber tRNA suppressor, although the overall protein synthesis was low in these systems. Another improved *E. coli* strain for NAA incorporation was obtained by knocking-out *prfA* gene combined with altering or “fixing” RF2 function using MDS42 [106] as the parental strain, in which the genome had been minimized through the removal of nearly 700 nonessential genes [103]. RF2 mutation T246A allowed it to be expressed constitutively in the host cell background and to increase the affinity of RF2 toward UAA codons, while A293E mutation almost completely rescued growth rate in RF1 knocked-out strain JX33 by weak termination of UAG stop codon [107]. The obtained JX33 strain was used for efficient incorporation of NAAs in response to multiple TAG sites. The partial and total genome reassignment was also applied for the creation of RF1-deleted strains. Seven essential *E. coli* genes (*coaD*, *hda*, *hemA*, *mreC*, *murF*, *lolA*, and *lpxK*) are terminated by amber codons. ORFs of all seven of these genes were engineered to end with the TAA stop codon, placed on a bacterial artificial chromosome (BAC), and the resulting BAC7 plasmid was introduced into an *E. coli* strain containing an amber suppressor tRNA. The *prfA* gene was then knocked out in these strains [108], which required the presence of both the amber suppressor and the BAC7 plasmid. Although the obtained strain was characterized by a slower growth rate, this RF1 knockout system was used for the incorporation of 3-iodo-L-tyrosine in response to seven amber codons. Similarly, the TAA termination codons were introduced into the end of seven essential genes by replacement of their genomic copies followed by an RF1 knockout [109]. Although the RF1 knockout in the resulting strain EcAR7 causes a decrease in the growth rate, it prevents premature stopping at the TAG codon and improves yields of phosphoprotein production. The B strain of *E. coli*, such as BL21(DE3), are widely used for the expression of recombinant protein under the T7-promoter regulation. The genome of BL21(DE3) contains 273 genes, including 95 essentials for growth of bacterial cells, are terminated by TAG stop codon. Using oligonucleotide-mediated recombination approach Mukai et al. replaced all 95 TAG codons on TAA or TGA stop signals and knocked out the *prfA* gene. The obtained B-95.ΔA strain demonstrated high efficiency in NAA-incorporated recombinant protein production without any attenuation in cell growth [110]. Finally, a new strain C321.ΔRF1 was evolved by replacement of all 321 known TAG stop codons in *E. coli* MG1655 with synonymous TAA codons, which permitted the deletion of RF1 and reassignment of TAG translation function. This genomically recoded organism (GRO) exhibited improved properties for the incorporation of NAAs in response to amber stop codon without any deleterious effect on the cellular functions [111]. The C321.ΔRF1 strain was demonstrated to be particularly useful for the multi-site incorporation of diverse NAAs [112]. 

**Figure 3:**
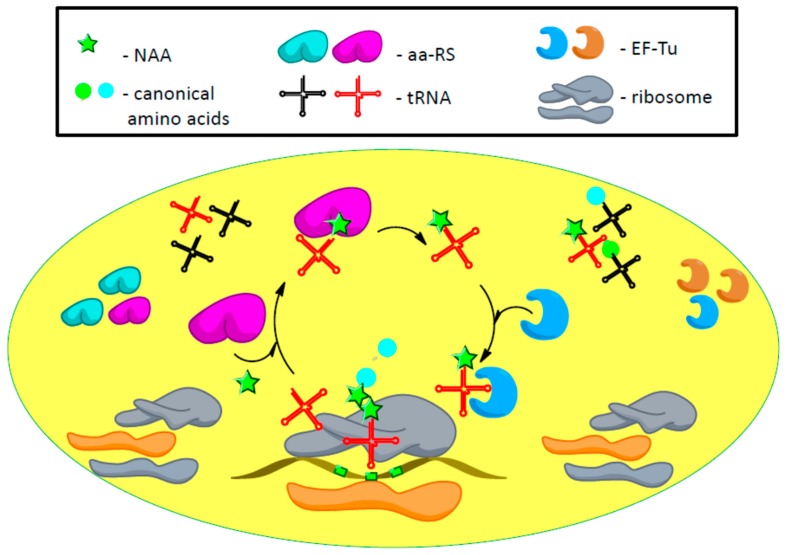
Improved orthogonal translation system includes aa-RS with enhanced enzymatic efficiency, optimized tRNA and evolved EF-Tu (shown by blue colour). Moreover, the overall efficiency of nonsense suppression is achieved due to the sequestration of RF1 from the translation process by application of orthogonal mRNA and 30S orthogonal ribosome subunit or by exploiting genetically recoded E. coli lacking RF1.

The summary of recently developed techniques applied to enhance efficiency of the genetic code expansion is illustrated by Figure 3.

## 4. Conclusions

The last decade was characterized by significant progress in the orthogonal translational system comprehension. Recent efforts in orthogonal translation systems improvement combined with the application of genetically recoded organisms allowed to improve the overall efficiency of methodology and to produce a recombinant protein with single or multiple site-specifically incorporated NAA with the yield equal or comparable with wild type protein synthesis. The intensive studies of the expanded genetic code methodology allowed not only for the development of highly effective tools for the protein molecular imaging, site-specific labelling, production of biomaterials or therapeutical peptide with improved and new properties [113], but also revealed that many other aspects must be considered as a potential limiting factor for the efficient NAA incorporation into recombinant proteins. For instance, the NAAs with bulky or extremely charged chemical group are unable to effectively cross cellular membrane, thus, the successful approach to evolve orthogonal pairs and site-specifically incorporate such a NAAs would be either propeptide strategy utilizing NAAs in dipeptide form [114] or modification of the host organism transport system, such as engineering of periplasmic binding protein, for optimized uptake of the desired NAA into the cell [115]. Very recently the significance of heterologous cellular background, particularly the enzymes modifying the orthogonal tRNA, for NAA-containing protein synthesis was demonstrated [116]. Another important issue to consider is the permissivity of the mutation site, i.e., sequence flexibility representing an ability to tolerate a large number of mutations at a selected locus without significant loss in protein activity [117]. The efficiency of NAA incorporation in response to a stop codon is known to depend on the position of the mutation site and the nature of the recombinant protein and the context dependence. The thorough analysis demonstrated [118] that mRNA, operon, and cellular context effects define the permissivity of the site for the NAAs incorporation. 

Despite the progress made in the NAAs incorporation field, it still has lots of challenges to be addressed in future investigation. For instance, although the repertoire of NAAs has been greatly extended, their diversity is still limited by the very few core structures defined mainly by the substrate specificity of evolved orthogonal synthetase. The proposed new approaches for the evolution of orthogonal pairs [79] and enhancement of their enzymatic activity [89] could be considered as a perspective to produce new orthogonal translation systems from other organisms and kingdoms that, in turn, can greatly increase the number and diversity of NAAs incorporation. Another challenging issue is the incorporation of multiple diverse NAAs in the same peptide or protein since they must be assigned by the different unique codons. This problem can be only partially overcome by the application of the frameshift suppression technique. However, the application of unnatural nucleotides and semi-synthetic organisms with two additional complement bases [119] made the number of codons assigned for the different NAAs is almost unlimited.

To summarize, the efficacy and the robustness the genetic code expansion methodology provides great opportunities in protein engineering both for the fundamental researches and for the applied biotechnology. Considering recent achievements and perspectives it might become the most powerful tool for the great area of application in protein science.
